# Ricord on Venereal Diseases

**Published:** 1846-03-01

**Authors:** 


### Ricord on Venereal Diseases.

Continued from page 212.

In our last number we presented to our readers a brief summary of the more important of the practical views and precepts of Ricord in relation to Gonorrhœa, as developed in a work by himself on Venereal affections, and in an English treatise by Acton, who prosecuted his observations under Ricord's instructions at the Venereal Institutions of Paris. We purpose continuing the analytical review of these works in the present article, premising, as before, that the limits to which we feel bound to adhere, preclude so full and satisfactory an exposition as we could desire, and that we shall endeavor to select what will be practically useful to the practitioner. Our remarks in the present number will be devoted to the class of Venereal complaints included under the name of *Syphilis.*

The questions whether there exists an identity of essence between Gonorrhœa and Syphilis, and whether they are under any circumstances mutually convertible, have been frequently discussed by medical writers. The experiments which John Hunter recounts of inoculation, however, would seem to settle this point, and demonstrate a radical distinction between the two diseases. He adduces numerous instances in which the matter of gonorrhœa was applied between the glans and prepuce, and introduced into the cellular tissue, without resulting in a single case in the development of a chancre; on the other hand, the matter secreted by a syphilitic ulcer was found frequently to reproduce, by inoculation, ulcers having the same specific characters.

This method of investigating the subject, viz: by inoculation, has been practiced by several observers, but with results which apparently conflicted with each other, and, thence, failing to afford any satisfactory conclusions beyond the distinction just stated. To Ricord is due the merit of having prosecuted experiments of this nature with much philosophical sagacity, and deduced certain facts which appear to settle the pathology on a firm basis, and which are susceptible of several important practical applications. A large portion of Ricord's work is devoted to the numerous observations of the facts connected with inoculation, his inductions therefrom, and their practical applications. We must pass over this portion very cursorily, much more so than we should desire, if, by extending our analysis we should not encroach on what is of greater practical import. We proceed to present, briefly, his general conclusions:

Pus taken from the surface of an ulcer *while the ulcer is progressive or stationary*, will give rise to a specific chancre, when inoculated in the same individual, with great certainty. This will not be the case after the ulcer begins to take on a reparative action, either spontaneously, or from the application of remedies. The omission to recognize the conditions of success which we have italicised, has been one cause of uncertainty and confusion attending previous experiments. The matter from gonorrhœa never produces similar results unless there co-exist a concealed chancre within the urethra. Ricord explains by this complication those anomalous cases in which gonorrhœa has appeared to give rise to Syphilis.—The appearances presented by inoculation of the chancre-pus we should be glad to quote if our limits permitted, but we must refer the reader to the description contained in either of the works referred to. Inoculation, therefore, while it demonstrates the existence of a *specific virus*, radically distinguishing Syphilis from other Venereal affections, furnishes an unfailing test by which the existence of the disease may be determined.—In any case of doubt or uncertainty we have only to introduce some of the pus into the cellular tissue of the same individual, (the thigh or arm are usually selected) and if a true chancre be the result, the question is settled. If no specific result follows, *provided the primary ulcer be progressive or stationary*, we have conclusive evidence that the disease is *not* Syphilitic.

It is fairly deducible from the results of inoculation, also, that the primary chancre involved the previous contact of a specific virus. By analogy it would follow that a sore thus reproducing by its products a similar sore wherever these were applied, must have been originally derived from a prior sore of the same character. Hence, we are to regard this disease transmissible, and extending itself only by the application and production of a specific morbid virus. An important inquiry follows: Are the consecutive affections following chancre capable of propagating the disease by inoculation? Ricord has applied this test, in the first place, to Buboes. He confirmed by his experiments the observations previously made, that the pus of buboes sometimes developed chancres by inoculation, and sometimes not: but by continuing his researches, he was led to the following inferences, which reconcile facts before unintelligible because apparently inconsistent with each other. He recognizes two kinds of buboes; one in which the inflammation and suppuration are merely sympathetic, proceeding, as they sometimes do, from other causes of irritation—for example from gonorrhœa; the other in which the virus is carried, by means of the lymphatics, from the chancre to the glands of the groin, and there being deposited, produces specific effects precisely as if by inoculation. In the former case, when the discharged pus is inoculated, negative results follow. In the latter case, a specific chancre is the result. Sometimes both of these forms of bubo co-exist even in the same groin. Ricord has met with cases of suppuration where an abscess being opened no results followed inoculation. At the bottom of the superficial abscess, however, another small collection of pus was found, which being opened, and inoculation practiced, a syphilitic ulcer was produced. These results are certainly highly interesting. A bubo produced by the absorption of virus, Ricord, in effect, includes among the *primary* symptoms. Acton applies to it the title of *internal chancre*.

A fact is stated by the latter Author with reference to the location of chancres as determining the development of buboes, which the practitioner will do well to bear in mind. It is, that when located at the inferior portion of the prepuce, and especially near the frœnum, buboes almost invariably follow. The explanation of this is, that the *vasa afferentia* terminating in the lymphatic glands of the groin, originate here in great abundance.

Inoculation is made by Ricord the test of the distinction between the *primary* and *secondary* symptoms. Numerous experiments were made by him to ascertain if the morbid products of secondary affections—such as the pus secreted by ulcerations of the throat, &c , would produce syphilitic ulcers, but all were followed by negative results. This, therefore, leads to a fundamental distinction—but of this more appropriately presently.

Experiments were made to determine if the syphilitic virus would be efficacious after the lapse of several days from the time when it was taken from the chancre. He found that after being kept eight days in tubes similar to those for the preservation of vaccine it would suffice for inoculation. Nevertheless, he thinks, to quote his language, that "*the fluid which serves as a vehicle for the virus ought to possess a certain degree of warmth, or kind of life, which gives the virus the power of attaching itself to the new bodies to which it has been transmitted.*" He confesses his incredulity as to contagion by means of privies, commodes, and clothes previously worn by those affected, which, if we always placed implicit confidence in the veracity of our patients, enter very largely into the etiology of this disease! He states, and adduces instances to prove that the virus may be communicated in sexual intercourse, by either sex, without the individual communicating it being affected—the organs in either case serving simply as mediums of transmission from an affected person to others.

The question could hardly fail to suggest itself to any one occupied in researches of this nature, whether something might not be discovered which would neutralize the virulent action of the syphilitic poison, and thus prevent the development of disease after exposure to infection had taken place. Accordingly we find that various experiments have been made with a view to the solution of this problem. Ricord recounts the experiments made by Luna Colderon in Paris, in 1845, in which, in presence of a medical commission, he subjected himself repeatedly to inoculation, in the first instance permitting the disease to continue until its characters had developed themselves sufficiently to demonstrate its existence, and after this, in the other trials, preventing the specific operation of the virus by certain remedies which he kept secret. The caustic alkalis, and weak acids, when mixed with the pus *before inoculation*, destroy its property of producing the disease; but these, nor aught else which Ricord has tried, are successful after the virus has once been introduced into the tissues. To quote his own expression of the results of his observations, "The efficacy of any of the above mentioned prophylactics cannot be depended upon for destroying the virulent pus which has even only just been put upon an entire surface, or momentarily to destroy a virulent secretion of an individual who could otherwise communicate disease." Nevertheless, Ricord expresses a confident anticipation that scientific investigations will "one day arrive at the discovery of an absolute neutralising substance for the specific principle of syphilis." Mere inoculation, of course, could not be expected to act in the light of a preservative, as in the case of variola, since it is well known that the disease may be contracted successive times with not less facility than at first.

It has been suggested that the artificial induction of this disease by inoculation may be useful in curing other affections; and, indeed, the trial has been made in several cases of protracted lues venerea, as it is stated with successful results. Ricord, however, does not concur in the propriety of subjecting a patient to the unforseen dangers incident to the induction of this disease, or to a new infection, while the benefit which he may derive is entirely problematical.

Finally, Ricord illustrates the application of inoculation to certain questions of a medico-legal character: for example, suppose an individual with gonorrhœa to be accused of committing rape upon a female who is found subsequently to have chancres; provided the gonorrhœa be not dependent on a concealed chancre, this coincidence cannot be regarded as evidence of his guilt, which it might be considered to be, had not inoculation demonstrated the inconvertibility of the two affections; and as regards the supposition of concealed chancre, inoculation enables this point to be satisfactorily determined.

With this general view we leave the subject of inoculation. The views of Ricord as connected with this subject, involve, to a considerable extent, his general opinions on the pathology of the disease. We will add somewhat, however, to the development of the latter before passing to the subject of treatment. Ricord considers the disease divisible into five forms, or, rather, he makes five classes of the symptoms more or less directly connected with syphilis. The *first* class embraces the *primary* affection, which is chancre from the direct action of the virus. The *second* class embraces the affections immediately succeeding (successive symptoms) as new chancres produced by direct extension of the virus from the first (a kind of involuntary inoculation;) and buboes, furnishing healthy, or virulent pus. We would here remark with respect to the occurrence of buboes not preceded by chancres, that in cases where such appears to be the fact, Ricord attributes their origin (if they are truly syphilitic, as determinable by inoculation) to chancres which escaped observation. Frequently it has occurred to him to discover on examination chancres, the existence of which the patient had never suspected, and if not discoverable, they may have existed and spontaneous cure taken place. The latter is not of very infrequent occurrence. The *first* and *second* classes, it will be observed, include affections in which a virus capable of inducing specific effects on inoculation is reproduced. The *third* class embraces what are ordinarily termed the *secondary symptoms* —symptoms expressive of a general infection of the system. Concerning the mode in which the constitution becomes affected, and the pathology of that general condition which precedes the local development of secondary symptoms, Ricord does not advance any speculations; and speculations are all that can be advanced on this subject in the present state of our science. He designates this constitutional state the *syphilitic temperament*. The virus in being introduced into the system, (if it be introduced) and producing this temperament, must undergo important modifications; for the secondary, local affections do not reproduce it in any form susceptible of producing specific effects by inoculation. Non-inoculability, as already mentioned, is made a characteristic of the *secondary*, as distinguished from *primary* symptoms. The secondary symptoms are capable of being transmitted by inheritance from mother to child, the latter "presenting after birth general symptoms corresponding to those of the mother, without previous primary affection." This is a characteristic of the class of secondary symptoms, as distinguished from the classes which follow.

These symptoms may develope themselves on the skin, the mucus membrane, eyes, testicles, &c. The particular characters which distinguish them, and the phenomena connected with them, our limits compel us to pass by. According to the observations of Ricord they seldom occur before the first two weeks of the duration of the primary affection, and, generally, after the *fourth*, *sixth*, *eighth*, or even much later. His observations, also, lead him to the conclusion that they rarely occur when the chancre does not have an indurated base, and edges, (the Hunterian chancre) and that they are rarely absent if these characters are present.

The *fourth* class embraces the *tertiary* symptoms These occur at indefinite periods, generally long after the cessation of the primary affection, and are usually absent unless preceded by secondary symptoms. They are not inoculable, nor hereditary. Under this head he includes nodes, deep-seated tubercles, periostosis, exostosis, caries, necrosis, &c. These denote a condition of the system which is often considered scrofulous, which is in such cases but "*degenerated Syphilis*." They indicate a general cachexy, developed by the operation of the Syphilitic poison; but in which the specific character of the virus is still more remotely operative than in the class of symptoms last mentioned.

Lastly, the *fifth* class embraces other affections in no wise connected with Syphilis, but the development of which has been favored by it—such as cancer, phthisis, scrofula previously existing, scurvy, &c.

We will now enter upon the *treatment*, to which the remainder of the article will be devoted. On this, as indeed every branch of the subject, the style of Ricord's treatise is so condensed, that it is impossible to give an abstract which will do justice to it. He says no more than he deems important, and that in the fewest possible words. We must confine ourselves to a brief account of the general principles of his practice, omitting, for the most part, particular modifications. He treats, first, of the treatment of chancre.

When this is presented in the form of a pustule or a small follicular abscess (two forms of the incipient development of the disease) he immediately punctures, evacuates the contents, and cauterizes with a sharply pointed stick of the nitrate of silver. If an ulcer be present when the disease is first examined, (as is generally the case) he resorts at once to cauterization, which he repeats until the surface takes on distinctly a healing action. In addition to this, in ordinary cases he makes use of dry lint to absorb the secretion, which he directs to be frequently changed, or lint dipped in the aromatic wine of the French Pharmacopeia. The object of the latter directions is to prevent the virus from accumulating, or extending the disease by contact with the neighboring parts. For the first five days after infection he apprehends not the least danger of constitutional infection. He states that there is not an authenticated instance in which this has happened. On this topic, he says "I have found in the subjects affected with constitutional syphilis who have come under my observation that the chancres had never lasted less than ten, twelve, or fifteen days, and in the majority their existence had been prolonged to two, three, four, five and six weeks, and longer."

When the tissues surrounding the chancre are engorged, or when it has acquired considerable extent the nitras argenti is not sufficiently energetic. In such cases he employs the caustic potash, or the Vienna paste, (equal parts of caustic potash and quicklime moistened with alcohol.)—These are of course to be applied with caution.

The object of these local applications is to destroy as speedily as possible the specific character of the ulcer, preventing the secretion of virulent pus, from which successive symptoms and constitutional infection follow, and inducing a condition favorable to reparation.

When the chancre assumes a phagedænic character, he directs his attention to circumstances which may induce or promote this tendency irrespective of the syphilitic disease, which may be some visceral derangement, a cold, damp dwelling, &c. After bestowing proper care upon these, and using nitrate of silver, emollients, anti-phlogistic narcotics, if the chancre still remains stationary or progressively advances, he either applies to it a blister, or sprinkles it with cantharides. If it succeeds a bubo he frequently fills the suppurating cavity with powdered cantharides, afterward dressing with the aromatic wine—in lieu of the wine Acton uses a combination of decoct. Querc. and tr. Catechu. The Cantharides may be renewed every three or four days until granulations appear.—Indurated chancre he deems deserving very particular care. Cauterization with the nitras argenti cannot be relied upon to cure the ulcer because it does not extend its influence beyond its limits, its local effect is however generally salutary. A favorite application is a combination of calomel and opium combined with cerate. But in this form of the disease he relies very much on the internal administration of mercurials. We have purposely avoided alluding to this remedy before in order to present the general views of Ricord as applicable to the various forms which the primary symptoms may assume. He evidently considers mercury quite unnecessary so long as there is no danger of constitutional infection from absorption. In the phagedænic form, in most cases he regards its operations prejudicial, yet in some instances it exerts good effects. He does not therefore resort to it unless other measures fail. Also where the phagedænic ulcer proceeds to a gangrenous form of inflammation (a variety we have not before alluded to) he says the worst results sometimes follow the empirical use of mercury. These cases are to be treated for the peculiar form of inflammation which they involve, overlooking for a time the primary cause. Afterward the chancre is to be treated upon the ordinary principles.

If we rightly apprehend his views then, he does not believe mercury to be required, and frequently, if not generally contra-indicated, excepting in cases of *indurated chancre*. This form of chancre our readers will recollect is observed to be the form generally preceding constitutional infection.

In bringing the system under the influence of mercurials, he pursues the mildest possible means. He usually gives the *proto-iodide* in one grain doses with *Hyoscyamus* at night, five hours after the last meal. It is to be continued until the induration ceases and for some time afterward. He does not deem it necessary to salivate; and this result is extremely rare at the Venereal Hospital.

The use of mercury in syphilitic diseases is a matter of great practical importance. Its indiscriminate and excessive employment has doubtless been a source of great evils, and the serious consequences of this remedy have been often confounded with the effects of the disease. On the other hand the advocates of the non-mercurial plan have carried the matter to an opposite extreme. There is a *juste milieu* somewhere, which it is very desirable to ascertain. If the views of Ricord be correct, he has conferred great benefit upon this branch of practice by indicating the circumstances which denote the use of this remedy, and those under which its use is unnecessary if not always injurious. Our space will not admit of commentaries or we should have more to say on this head.

*Of the treatment of Buboes.*—The treatment for buboes at their commencement is directed to the prevention of their further progress. For this object absolute rest and the anti-phlogistic regimen are to be enjoined. In addition to these, Ricord employs, when it can be borne, methodic compression either by bandage or by a kind of truss devised for this purpose. This measure is, we imagine, but little used in this country. If this cannot be borne, or proves ineffectual, he covers the tumor with a blister, removes the epidermis, and places on the denuded skin lint dipped in solution of corrosive sublimate, 20 grs. to the ounce, which is allowed to remain two or three hours. Sulphate of Copper 2 or 3 drachms to the ounce, may be substituted for the corrosive sublimate. To obtain the desired effect an eschar must be produced penetrating part of the dermis. This method of treatment is only to be resorted to in buboes following chancre and where a virulent bubo is anticipated. Where they are symptomatic of gonorrhœa or merely sympathetic of chancre, he is satisfied to apply cold lotions, or cataplasms, leeches, sedative applications, and rest, in conjunction with general measures, such as saline cathartics and low diet.

When suppuration results notwithstanding the efforts made to prevent it, he advises to give exit to it so soon as its presence is positively determined. If it assume the character of chancre he applies Pulv. canthandes as already mentioned.

As regards the employment of the remedies called anti-syphilitic (of which mercury is the chief) they are indicated by precisely the same conditions as in chancre. The presence of a bubo does not constitute itself an indication for their use. Here the treatment is deducible from the pathology which the reader will recollect. When they are conjoined with the lymphatic or scrofulous habit, he has found great advantage from the proto iodide of iron in doses of ten, twelve, to twenty grains per diem, in a decoction of hops, or syrup of Gentian.

*Of the treatment of constitutional Syphilis.*—This we must dismiss with great brevity, not only because we have already occupied as much space as can well be devoted to this subject, but it would be impossible to go into any detail without taking up the various forms of secondary and tertiary symptoms seriatim, which would involve a very extended analysis. We shall confine ourselves to his general principles as applicable to the various affections included under the classes called secondary and tertiary, omitting all minor, adjunctive matters. He relies upon mercury in Secondary Syphilis when not specially contra indicated by circumstances not connected with the Syphilitic disease. He employs generally the proto-iodide in the same way as in chancre. Introduced endermically he deems an inferior method, excepting when the bad condition of the digestive organs will not admit of the more direct method. He is equally careful to avoid salivation, always regarding this accident as an evil.

Sudorifics he thinks have been too much lauded. They are indicated when mercury cannot be administered or when it has produced ill effects. But it is chiefly as a *moral* medication that they are useful, owing to the popular confidence placed in them. In that condition of which every practitioner has seen specimens, which our author terms *Syphilophobia*, in which the patient is haunted by the idea that the disease is lingering in the system, this class of medicines may be prescribed under the appellation of correctives, depuratives, or, vulgarly, *purifiers of the blood.* He gives the first place to Sarsaparilla. Guiacum has succeeded best in affections of the osseus system. When practicable these remedies should be given in the form of *tisanne* or diet drink.

This treatment relates to the *secondary* symptoms. In the tertiary affections the pathology is widely different. In the secondary form, to quote his language, the "virulent cause still exists; in the tertiary it is completely transformed." The principles of treatment are in correspondence with this view of the pathology. The specific proximate cause—whatever it be—no longer present as such in the system, the specific efficacy of mercury is not required as an anti-syphilitic. These affections are then to be treated upon general principles. Mercury is usually positively prejudicial. In the place of this the *iodide of potassium* is to be employed, and this may be resorted to with as much confidence in the tertiary as mercury in the secondary symptoms. The following is Ricord's method of using this remedy, which we find quoted in the work of Acton. "We may commence with a dose of ten grains taken daily in a mixture, for which I give the following formula: Distilled water, ℥ iii. Iodide of Potassium, gr. x. Syrup of Poppies, ℥ i. This mixture should be taken in the course of the day in three glasses of decoction of Sarsaparilla, or some bitter infusion. The dose may then be augmented by ten grains every five days until the dose of a hundred grains may be taken daily: this dose I have rarely exceeded."

The use of this medicine sometimes produces pains or uncomfortable sensations in the stomach, small pustules on the skin, diuresis, and iodic intoxication, characterised by a slight uncertainty in the voluntary movements, some subsultus tendinum, heaviness in the head, a species of intellectual idleness and sometimes slight delirium.

In closing this analysis we must confess that before the task was half performed, we had regretted its commencement, finding that within the limits to which we were restricted it was quite impossible to do justice to the works and to the subject. Our readers, however, will of course appreciate the necessity of passing over all points with a brevity inadequate to their merits, of omitting entirely many topics of more or less interest and importance, and of repressing the disposition to add to the length of the article by commentaries of our own.

The work of Ricord embraces an appendix containing a list of the formulas of the various medicaments used in the wards of the *Hospital des Vinireris*, which for the practical reader is especially valuable.

