# Erratum

**Published:** 1846-03-01

**Authors:** 


*Erratum.*—For Hospital des Vinireris, page 225, read *Hopital des Veneriens.*

